# Using the visual arts to teach clinical excellence

**DOI:** 10.15694/mep.2018.0000143.1

**Published:** 2018-07-16

**Authors:** Eden Gelgoot, Christine Caufield-Noll, Margaret Chisolm

**Affiliations:** 1 McGill University; 2 Johns Hopkins Bayview Medical Center; 3 Johns Hopkins University School of Medicine

**Keywords:** Clinical excellence, Visual arts, Communication, Humanism and professionalism, Empathy, Observation

## Abstract

This article was migrated. The article was marked as recommended.

**Introduction:** The authors conducted a review of the literature to identify curricula that incorporate the visual arts into undergraduate, graduate, and continuing medical education to facilitate the teaching of clinical excellence.

**Methods:** The authors searched the PubMed and ERIC electronic databases in May 2017, using search terms such as “paintings,” “visual arts,” and “medical education,” along with terms corresponding to previously defined domains of clinical excellence. Search results were reviewed to select articles published in the highest impact general medicine and medical education journals describing the use of visual arts to teach clinical excellence to all levels of medical trainees and practicing physicians.

**Results:** Fifteen articles met inclusion criteria. Each article addressed at least one of the following clinical excellence domains: communication and interpersonal skills, humanism and professionalism, diagnostic acumen, and knowledge. No articles described the use of the visual arts to teach the skillful negotiation of the health care system, a scholarly approach to clinical practice, or a passion for patient care.

**Conclusions:** This review supports the use of visual arts in medical education to facilitate the teaching of clinical excellence. However, research designed specifically to evaluate the impact of the visual arts on clinical excellence outcomes is needed.

## Introduction

Medical schools are increasingly recognizing the role of the arts and humanities in the professional formation of clinically excellent physicians ( Rodenhauser, Strickland and Gambala, 2004). The arts and humanities allow trainees to explore the diversity of the human experience and to reflect on an individual patient’s experience with illness or grief ( Mullangi, 2013). Exposure to the humanities has also been correlated with reduced burnout among medical students ( Mangione *et al.*, 2018). A recent systematic review on the use of the creative arts in health profession education found these curricula promote learner engagement, foster the discovery and creation of meaning, and can lead to better medical practice ( Haidet *et al.*, 2016). Thus, medical schools are increasingly interested in ways to integrate the arts into their curricula, and some have already done so ( Bramstedt, 2016). Although there has not been a recent systematic review of the literature specifically focused on the incorporation of visual arts into medical school curricula, a 2002 survey of arts-based programs at U.S. medical schools found that the visual arts had been incorporated into 18 required courses, 36 elective courses, and 29 extracurricular activities, among the 83 medical schools that responded to the survey ( Rodenhauser, Strickland and Gambala, 2004).

The integration of the visual arts into medical education serves both explicit and implicit functions ( Bardes, Gillers and Herman, 2001). Explicitly, the visual arts assist in the development of clinical skills, including the observation, analysis and communication of visual information ( Bardes, Gillers and Herman, 2001; Shankar, Piryani and Upadhyay-Dhungel, 2011). Implicitly, the visual arts add a subjective dimension to the objective study of the pathophysiological model of disease, helping students to recognize the individual patient’s experience with illness instead of viewing them as “elaborate machines” ( Bardes, Gillers and Herman, 2001). This subjectivity challenges students’ discomfort with ambiguity, encourages them to confront their own emotions, disrupts assumptions, and fosters an awareness of multiple perspectives ( Haidet *et al.*, 2016; Kumagai, 2017). Representational art allows learners to focus on identifying recognizable forms and contextual information; while abstract art fosters the development of pattern recognition skills, fosters increased tolerance of ambiguity, and provides learners with the freedom to follow their own imagination and emotions ( Jasani and Saks, 2013; Karkabi, Wald and Cohen Castel, 2014).

Although medicine is considered to be in part a visual science, physicians often miss the importance of “seeing patients” ( Boisaubin and Winkler, 2000). The visual narratives represented in paintings can assist in developing insight into the less obvious aspects of a patient’s experience ( Arnold, Lloyd and von Gunten, 2016), encouraging the exploration of the human dimensions of illness and suffering ( Kumagai, 2012).

Achieving clinical excellence involves mastery of the “art” of medicine ( Christmas *et al.*, 2008). Cognizant of the value of such clinical mastery, the Johns Hopkins School of Medicine established the Miller-Coulson Academy of Clinical Excellence (MCACE) to recognize and reward the work of clinically excellent physicians ( Christmas *et al.*, 2008; Wright *et al.*, 2010). Based on a systematic review of the literature and qualitative research, the MCACE defined clinical excellenceas achieving a level of mastery in the following domains as they relate to patient care: (1) communication and interpersonal skills, (2) professionalism and humanism, (3) diagnostic acumen, (4) skillful negotiation of the health care system, (5) knowledge, (6) scholarly approach to clinical practice, (7) exhibiting a passion for patient care, (8) explicitly modeling this mastery to medical trainees, and (9) collaborating with investigators to advance science and discovery ( Wright *et al.*, 2010).

In the present paper, the authors hypothesized that the incorporation of the visual arts into medical education curricula could be used to teach clinical excellence. To test this hypothesis, the authors conducted a review of the literature to identify examples – drawn from some of the highest impact general medicine and medical education journals – of curricula that use the visual arts in undergraduate (UME), graduate (GME), and continuing medical education (CME) curricula to teach clinical excellence.

## Methods

One of the authors (CC-N), a medical informationist, designed and executed a search of the PubMed and ERIC electronic databases in May 2017 to identify a body of published articles relevant to the topic of interest. Controlled vocabulary and keyword terms including “paintings,” “visual arts,” and “medical education” were combined with terms corresponding to each domain of clinical excellence, as defined by the MCACE ( Wright *et al.*, 2010). The authors excluded domains 8 and 9, “explicitly modeling this mastery to medical trainees” and “collaborating with investigators to advance science and discovery,” since these domains are limited to clinicians working in academic settings. Search results were refined by date (2000-present), publication type (journal article), and language (English only), and then limited to a predetermined subset of 40 of the highest-impact general medicine and medical education journals ( Appendix 1) to achieve a snapshot of the key literature on this topic. Journal impact factors were determined by searching the 2016 edition of Journal Citation Reports® (Clarivate Analytics, 2016). Retrieved citations were exported to RefWorks reference management system for organization, deduplication, and title and abstract scanning.

One of the authors (EG) reviewed the articles and selected those that met the following inclusion criteria: describes required and/or elective medical education curricula incorporating the examination of paintings (representational or abstract) and/or the creation of original artwork for learners at the UME, GME, or CME level. Duplicate titles, those with clearly irrelevant subject matter, and those for which the full text was not available were excluded from full-text review. Each article advancing to full-text review was read and summarized by one of the authors (EG), with particular focus on descriptions of curricula and, when applicable, the outcome measures used to evaluate impact.

In addition, EG assessed each curricula as to the level of its outcomes (reaction, learning, behavior, and results) using the Kirkpatrick’s Model ( Kirkpatrick and Kirkpatrick, 2006). Level 1 of the hierarchy (reaction) measures “the degree to which participants [found] the training favorable, engaging and relevant”; Level 2 (learning) measures the degree “to which participants change[d] attitudes, improved[d] knowledge, and/or increase[d] skills”; Level 3 (behavior) measures the degree to which participants “change[d] their behavior” once they were back on the job; and Level 4 (results) measures the degree to which targeted outcomes occurred ( Kirkpatrick and Kirkpatrick, 2006).

## Results

The search yielded 67 citations, 15 of which met the inclusion criteria.

**Figure 1.**
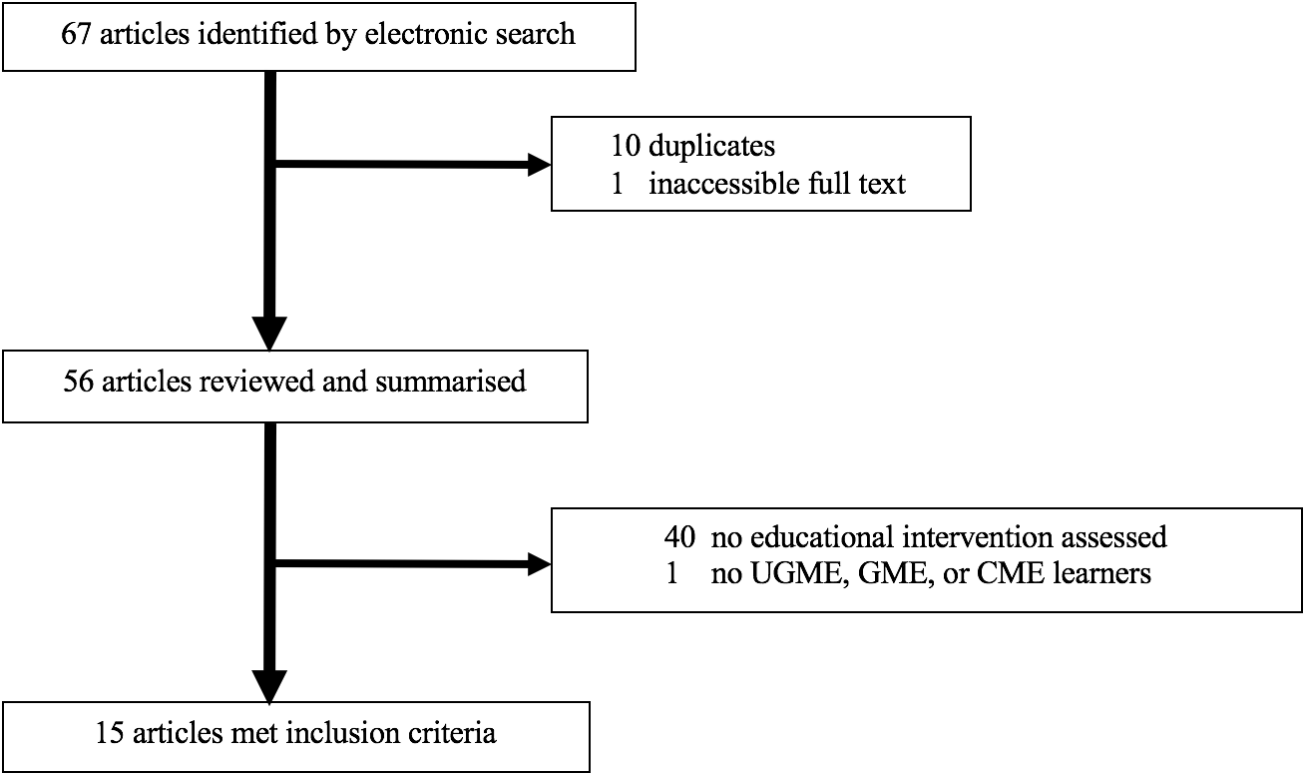
Flowchart for search strategy and review on use of visual arts to teach clinical excellence

The results of the review are summarized in Table 1.

**Table 1.** Summary of articles identified on use of visual arts to teach clinical excellence

Domain of clinical excellence	Authors, year	Targeted learners; Institution, Country	Intervention	Examples of visual art used in activity
Communication and interpersonal skills	Boisaubin and Winkler, 2000	1st year medical students; University of Texas Medical Branch, United States	Required weekly semester-long course in which different representational paintings were used to spark reflection on topics such as mental illness, the doctor-patient relationship, death and dying, humanity and suffering, and physician-assisted suicide	Rembrandt, *Dr. Tulp’s Anatomy Lesson* Gericault, series of portraits Käthe Kollwitz, series of lithographs on the theme of death Norman Rockwell, *Saturday Evening Post* magazine covers
	Karkabi and Cohen Castel, 2011	4th year medical students; The Rappaport Faculty of Medicine, Technion Israel Institute of Technology, Israel	3-hour elective workshop in which students wrote narratives about the doctor-patient relationship based on paintings and first-account experiences	Francisco de Goya, *Self-portrait with Dr. Arrieta* Luke Filde, *The Doctor* Pablo Picasso, *Science and Charity*
	Kumagai, 2012	1st and 2nd year medical students; The University of Michigan Medicine, United States	The Family Centered Experience (FCE) is a required course during which students are matched with patient-volunteers with chronic illness and create original artwork based on their interactions with patients	Creation of original artwork
	Karkabi, Wald and Cohen Castel, 2014	Family physicians or physicians-in-training; 2012 World Organization of National Colleges, Academies, and Academic Associations of General Practitioners/Family Physicians (WONCA) Conference, Austria	Elective development workshop aimed at enhancing reflective capacity through combined viewing of abstract paintings and writing of short stories, to enhance understanding of human suffering. This article is also relevant to the domain of professionalism andhumanism	Mark Rothko, *Red and Orange* Jackson Pollock, *Full Fathom Five* Vassily Kandinsky, *Composition number 6*
	Arnold, Lloyd and von Gunten, 2016	Palliative care physicians; San Diego Hospice and the Institute for Palliative Medicine, United States	Required program to elucidate experiences with patients’ dying and death as “teaching moments” that result in positive personal and professional growth	Creation of original artwork
Professionalism and humanism	Karkabi and Cohen Castel, 2006	Family physicians; The Annual Meeting of the Israel Association of Family Medicine in 2004, Israel	2-hour workshop on “Suffering in the Mirror of Arts” designed to enhance understanding and deepen compassion for sufferers. Participants were shown 3 paintings and then required to write a short story about the paintings and to present these stories to the group	Rembrandt, *The Return of the Prodigal Son* Edvard Munch, *Death in the Sickroom* Sir Luke Fildes, *The Doctor*
	Winter and Birnberg, 2006	Residents; JFK Medical Center, Edison, United States	Required seminar that used open-ended questions to discuss the idea of professionalism in relation to 2 paintings depicting doctors in different clinical scenarios	Thomas Eakins, *The Agnew Clinic* Norman Rockwell, *Norman Rockwell Visits a Family Doctor*
	Shankar, Piryani and Upadhyay-Dhungel, 2011	1st year medical students; Manipal College of Medical Sciences and KIST Medical College, Nepal	Required bi-weekly program of 7-months duration that involed the analysis of paintings to improve the students’ empathy for the sufferer	Alice Neel, *City hospital* Jean-Baptise Grueze, *The paralytic* Vincent Van Gogh, *Portrait of Dr. Gachet*
	Yang and Yang, 2013	1st year postgraduate residents and clerks; The Changhua Christian Hospital, Taiwan	Required 4-hour program that involved the interpretation of paintings related to medicine, illness and human suffering	Not specified
Diagnostic acumen	Bardes, Gillers and Herman, 2001	1st, 2nd, and 4th year medical students; A collaboration between the Frick Collection and Weill Cornell Medical College, United States	Elective program aimed at developing skills in observation, description and interpretation, with a focus on the human face. All three sessions of the program took place at the museum. Session 1 (the “pre-test”) involved examining a patient’s photograph and then a painted portrait. The second session involved examining and presenting on a painted portrait. The third session (the “post-test”) involved noting comparisons and contrasts between patient photographs	Portraits by Western artists from the 16th and 19th centuries including Titian, Ingres, Rembrandt, and Gainsborough
	Dolev, Friedlaender and Braverman, 2001	1st year medical students; Yale University School of Medicine and the Yale Center for British Art, United States	The Yale Center for British Art (YCBA) project was an elective part of the doctor-patient encounter course and involved the observation and description of visual information in paintings at the museum	John Ferderick Lewis, *And the prayer of faith shall save the sick* J.M.W. Turner, *Dort or Dordrecht: The Dort Packet-Boat from Rotterdam Becalmed* Frederic Leighton, *Mrs. James Guthrie* Henry Wallis, *The Death of Chatteron*
	Miller, 2010	2nd year medical students; A collaboration between Columbia University College of Physicians and Surgeons and the Frick Collection, United States	Required class aimed at sharpening observational skills through the viewing of a painting for 5-minutes and group discussions on students’ observations	Giovanni Bellini, St. Francis in the Desert
	Jasani and Saks, 2013	3rd year medical students; Rutgers Robert Wood Johnson Medical School, United States	Required 3-hour exercise that included the discussion of 8 fine art images using the VTS questions with the aim of enhancing clinical observation skills in clinical diagnosis	Not specified
	Bramstedt, 2016	Medical students; Bond University Medical School, Queensland, Australia	The medical humanities curriculum involved: (1) an elective Medical Humanities Workshop which teaches VTS, (2) a required mixed media art creation on any topic related to health care and a reflective essay assignment, (3) Medical Humanities Week, and (4) the Art is Good Medicine community art exhibit during which their work is showcased	Creation of original artwork Lorenzo Valles, *Madness of Joanna of Castile* Francisco de Goya, *Self-portrait with Dr. Arrieta* Giovanni da Fiesole, *Healing the Deacon Justinian*
Knowledge	Finn, White and Abdelbagi, 2011	1st year medical students; School of Medicine and Health, Durham University, United Kingdom	One-hour elective course that used body painting to teach anatomy	Not specified

According to our assessment of each article using the Kirkpatrick’s Model ( Kirkpatrick and Kirkpatrick, 2006), 7 articles ( Boisaubin and Winkler, 2000; Karkabi and Cohen Castel, 2006, 2011; Shankar, Piryani and Upadhyay-Dhungel, 2011; Kumagai, 2012; Karkabi, Wald and Cohen Castel, 2014; Bramstedt, 2016) describe interventions that measured outcomes at the level of learner reaction. Six articles ( Bardes, Gillers and Herman, 2001; Dolev, Friedlaender and Braverman, 2001; Finn, White and Abdelbagi, 2011; Jasani and Saks, 2013; Yang and Yang, 2013; Arnold, Lloyd and von Gunten, 2016) describe interventions that measured outcomes at the level of learning. No articles were identified that evaluated interventions at the level of learner behavior or results.

What follows is a summary of key findings of the 15 articles identified by this review on the use of visual arts to teach clinical excellence, organized by the MCACE-defined clinical excellence domain.

### Communication and interpersonal skills

Communication and interpersonal skills are fundamental to clinical excellence ( Christmas *et al.*, 2008). Being able to form strong relationships with patients, be team players, remain flexible, and simplify medical concepts to ensure patient understanding play a major role in how the public perceives physicians ( Doukas *et al.*, 2015). In addition, reflective capacity, defined as “the critical analysis of knowledge and experience to achieve deeper understanding, guiding future behavior,” is essential for effective communication, positive physician-patient relationships, and the accurate collection of clinical information ( Karkabi and Cohen Castel, 2011; Karkabi, Wald and Cohen Castel, 2014). Reflection is seldom intuitive for learners or teachers, thus the determination of how best to teach reflection in medical education is critical ( Karkabi and Cohen Castel, 2011; Karkabi, Wald and Cohen Castel, 2014). The integration of the visual arts into medical education supports the development of reflective capacity, by providing a space to contemplate difficult issues, such as what it means to be a doctor, death and dying, and ethical dilemmas ( Rodenhauser, Strickland and Gambala, 2004; Karkabi, Wald and Cohen Castel, 2014).

This review identified 5 articles that describe the use of visual arts to teach the communication and interpersonal skills domain of clinical excellence. Each of these articles described curricula whose goals are to increase reflective capacity of medical students in relation to the doctor-patient relationship ( Boisaubin and Winkler, 2000; Karkabi and Cohen Castel, 2011; Kumagai, 2012; Karkabi, Wald and Cohen Castel, 2014; Arnold, Lloyd and von Gunten, 2016). The first describes a semester-long weekly UME curriculum in which students discussed the themes depicted in representational artwork as they relate to the practice of medicine ( Boisaubin and Winkler, 2000). Students reflected on an individual’s experience of mental illness through discussion of Gericault’s portraits of the “insane,” on body image and obesity through discussion of Ruben’s nude painting *The Fur (“Het Pelsken”)*, and on end-of-life care and physician-assisted suicide through discussion of Käthe Kollwitz’s lithographs on the theme of death.

The second article describes a three-hour UME workshop in which students viewed two paintings – Luke Fildes’ *The Doctor* and Pablo Picasso’s *Science and Charity* – each portraying scenes of a doctor-patient relationship ( Karkabi and Cohen Castel, 2011). Students wrote a first-person account from the perspective of a character in one of the paintings, followed by a reflection on their own patient encounters.

The third describes a longitudinal two-year UME curriculum that involved ongoing conversations between students and volunteer patients centered around what it is like to live with chronic illness and negotiate the health care system ( Kumagai, 2012). Students produced original artwork based on their interactions with those patients, the creation of which served three main purposes, to explore: (1) art as an *expression of identity,* tangible expressions of the students’ attempts to take the perspective of the patient, (2) art as *critique* of the status quo (e.g., power structures in health care, traditional doctor-patient relationships) and (3) art as *interpretation* of the conversations between students and patients.

The fourth describes a multinational CME curriculum in which practicing physicians first viewed an abstract painting like Rothko’s *Red and Orange* and Jackson Pollock’s *Full Fathom Five* ( Karkabi, Wald and Cohen Castel, 2014). This served as a visual prompt for a writing activity in which each learner was asked to reflect on a particularly challenging and/or meaningful clinical situation. Learners reported that the viewing of abstract paintings helped prepare them emotionally for reflective writing.

The fifth article describes a required CME curriculum, for which physicians who had recently completed a one-year fellowship in palliative care were asked to reflect on their experiences dealing with the death of patients and to create original artwork ( Arnold, Lloyd and von Gunten, 2016). Qualitative coding of 75 images revealed 2 categories of underlying visual metaphors (portraits and landscapes), representing the transient nature of life and death. The authors suggest that the visual narratives reflect a positive and hopeful viewpoint of death and dying (rather than ones associated with anxiety, pain, or suffering), which may be attributed to the development of personal and professional skills gained during the fellowship.

### Professionalism and humanism

The MCACE defines professionalism and humanism as generosity with patients and with one’s time, being honest, non-judgmental and caring ( Christmas *et al.*, 2008). Historically, medical education has taught professionalism through lectures and clinical vignettes ( Winter and Birnberg, 2006). However, learners who have participated in arts-based courses report that this method of learning helped them develop professionalism skills. In addition to professionalism, humanism – the recognition of each patient as a person of inherent value – is essential to clinical excellence. However, developing medical learners’ compassion for their patients is an ongoing challenge in medical education ( Karkabi and Cohen Castel, 2006). Empathy - the ability to understand the perspective of the patient and to communicate this understanding with the patient - is central to humanism ( Yang and Yang, 2013). Paintings can serve as mirrors of the human condition and have been shown to deepen appreciation of human suffering and enhance empathy for patients among medical learners ( Karkabi and Cohen Castel, 2006).

This review identified 4 articles that describe the use of visual arts to teach the professionalism and humanism domain of clinical excellence ( Karkabi and Cohen Castel, 2006; Winter and Birnberg, 2006; Shankar, Piryani and Upadhyay-Dhungel, 2011; Yang and Yang, 2013), three of which specifically describe curricula which incorporated the visual arts to better understand the nature of human suffering and to deepen compassion for sufferers ( Karkabi and Cohen Castel, 2006; Shankar, Piryani and Upadhyay-Dhungel, 2011; Yang and Yang, 2013). The first article describes a 2-hour CME curriculum which involved the examination of 3 paintings (Rembrandt’s *The Return of the Prodigal Son,* Edvard Munch’s *Death in the Sickroom,* and Sir Luke Filde’s *The Doctor),* writing a short story about the paintings, and presenting these stories to the group ( Karkabi and Cohen Castel, 2006). The 18 participants who provided feedback at the end of the curriculum indicated a change in their attitudes towards compassion for the sufferer, and a positive sentiment towards the activity.

The second article describes a GME seminar on professionalism during which Thomas Eakins’ *Agnew Clinic* and Norman Rockwell’s *Norman Rockwell Visits a Family Doctor* were used to guide discussions on the meaning of professionalism ( Winter and Birnberg, 2006). Residents were asked open-ended questions about the professional behaviors portrayed in each of the paintings and to compare these behaviors to those described by the Association of American Medical Colleges and the National Board of Medical Examiners. Residents reported that this curriculum engaged them emotionally, fostering discussion of the professional ideals that first inspired them to pursue medicine as a career. They also reported that this creative approach to teaching was a more effective method than didactic lectures and isolated clinical vignettes in teaching professionalism.

The third article describes a required bi-weekly 7-month UME curriculum in Nepal involving the analysis of paintings such as Alice Neel’s *City hospital* and Vincent Van Gogh’s *Portrait of Dr. Gachet* to improve empathy in learners for the sufferer ( Shankar, Piryani and Upadhyay-Dhungel, 2011). Students reported that they had difficulty extrapolating the context depicted in Western paintings to Nepal and that they would have preferred a course based on paintings by Nepalese artists that better reflected their own experiences. 29.5% of respondents (n=23/78) believed that the incorporation of the visual arts in medical education promotes empathy.

The fourth article describes a required 4-hour GME curriculum involving the interpretation of paintings related to medicine, illness, and human suffering, and which used the Jefferson Scale for Physician Empathy (JSPE) to measure the quantitative effects on empathy ( Yang and Yang, 2013). No significant differences between the pre-test and post-test JSPE scores were measured, which the authors suggest may be attributed to the small sample size (n=110) and the fact that the duration of the workshop was only four hours.

### Diagnostic acumen

Physicians considered to be “skillful diagnosticians” are thorough, exercise outstanding judgment, and are often called to solve puzzling cases ( Christmas *et al.*, 2008). Being a skillful diagnostician requires mastery of the observation, description, and interpretation of visual information, skills often considered the “special province of the visual arts” ( Bardes, Gillers and Herman, 2001). In the visual arts, the “art of looking” is made explicit through an emphasis on the intense and detailed observation and description of visual information ( Bardes, Gillers and Herman, 2001; Doukas *et al.*, 2012). A detailed examination of paintings can teach medical learners this skill of “slow looking,” and assist them to distinguish between primary observable and confirmable visual information vs. secondary and derived inferences ( Bardes, Gillers and Herman, 2001). In the medical field, *visual literacy* has been defined as “the capacity to identify and analyze facial features, emotions, and general bodily presentations, including contextual features such as clothing, hair and body art” ( Bramstedt, 2016). Heightened visual literacy can assist physicians in reaching a diagnosis, making the invisible visible ( Wellbery and McAteer, 2015; Bramstedt, 2016) and is particularly valuable in situations when patients are unable to communicate their symptoms ( Bramstedt, 2016).

This review identified 5 articles that describe the use of visual arts to teach the diagnostic acumen domain of clinical excellence ( Bardes, Gillers and Herman, 2001; Dolev, Friedlaender and Braverman, 2001; Miller, 2010; Jasani and Saks, 2013; Bramstedt, 2016). The first article describes a UME curriculum facilitated by a collaboration between an art museum and a medical school ( Bardes, Gillers and Herman, 2001). The program consisted of three sessions during which students focused on the evaluation of the human face as the pre-eminent expression, not only of health and disease, but also of emotion and character. Students examined painted portraits that were part of the museum’s collection including Titian’s *Portrait of a Man in a Red Cap,* Rembrandt’s *Nicolaes Ruts,* and Ingres’s *Comtesse d’Haussonville.*

The second article describes a similar UME curriculum that aimed to teach observational and descriptive skills through the analysis of the museum’s paintings ( Dolev, Friedlaender and Braverman, 2001), such as Henry Wallis’s *The Death of Chatteron* and J.M.W. Turner’s *Dort or Dordrecht: The Dort Packet-Boat from Rotterdam Bacalmed* ( Yale School of Medicine, 2015). Discussion was facilitated using the following guiding questions: “Is the figure sleeping?”; “Where in the house is this scene located?”; “What is the time of day?”; “How old is the figure?”; “What does his fisted left hand and arm position indicate?”; and “What was his cause of death?”

The third article describes a required UME curriculum that involved viewing paintings, such as Giovanni Bellini’s *St. Francis in the Desert,* and discussing observations in a museum setting ( Miller, 2010). The author reports on her own experience as a participant in the curriculum suggesting that it not only sharpened her observational skills, but that her role in the exercise was transformed from an *observer* to a “profoundly engaged *participant* in this work of art,” which she felt was facilitated by the non-judgemental group dialogue.

The fourth evaluated a three-hour long UME curriculum that was part of a required week-long course ( Jasani and Saks, 2013). A student researcher employed a four-step method for teaching clinical observational skills to students through the analysis of eight paintings: (1) *observation* of visual findings using the three visual thinking strategies (VTS) questions, (2) *interpretation* of the works, (3) *reflection* on the validity of their evaluations, and (4) *communication* of their ideas. *Visual thinking strategies* (VTS) is a technique used to enhance visual literacy in medical learners, using three questions to focus observations during the examination of paintings: “What do you see?”; “What makes you say that?”; and “What else do you see?” ( Jasani and Saks, 2013).

The fifth article describes a UME curriculum of an optional 50-minute workshop using the VTS questions to improve visual literacy, and a 7-week required mixed media art project and reflective essay on a health-related topic ( Bramstedt, 2016). Of the 66 individuals who completed the voluntary feedback survey, 54.6% supported the addition of arts education to the medical school curriculum. All 3 cohorts (2014-2016) of learners exposed to this arts-based curriculum reported reflective capacity as the skill which improved the most, followed by observational skills. However, 43.8% and 40.6% from cohort 1 (2014) and cohort 3 (2016), respectively, indicated that the curriculum had no impact on their skills.

Bardes, Gillers and Herman (2001), Dolev, Friedlaender and Braverman (2001), and Jasani and Saks (2013) compared observational skills within and/or among learners before and after exposure to curricular interventions. Bardes, Gillers and Herman found that medical students were more precise in their descriptions and able to make a greater number of inferences after exposure to the curriculum (the authors did not report whether this difference was statistically significant) ( Bardes, Gillers and Herman, 2001). In the pre-test, students described the features of a middle-aged woman portrayed in a photograph objectively with reference to her jewelry, features and make-up, while they made a greater number of inferences in the post-test describing the same photograph (e.g., the subject appeared “sad,” “worried,” “ill,” etc.). Dolev, Friedlaender and Braverman found no significant differences in pre-intervention test scores among individual medical students exposed to either a museum visit, an anatomy lecture, or a clinical tutorial session in both the 1998-1999 cohort and 1999-2000 cohort, while post-intervention scores differed significantly between groups in both cohorts ( Dolev, Friedlaender and Braverman, 2001). Dolev, Friedlaender and Braverman also found that the museum group – as a whole – had significantly higher post-test percentage improvement compared to the clinical and control groups in the 1998-1999 cohort, and a significantly higher post-test percentage improvement score compared to the clinical group in the 1999-2000 cohort (this cohort did not use a lecture group because preliminary data revealed no change in students’ observational performance). Jasani and Saks found that the mean number of observations between pre- and post-tests was not significantly different ( Jasani and Saks, 2013). Qualitative analysis revealed that in comparison to the pre-test written responses, the post-test responses showed decreased use of subjective terminology by 65%,such as “normal” or “healthy;” increased scope of interpretations by 40%; increased speculative thinking by 62%; and increased use of visual analogies by 80% after exposure to the curriculum (whether these differences were significant was not reported).

### Skillful negotiation of the health care system

This clinical excellence domain involves the health care systems in which physicians practice medicine, with excellent clinicians distinguished based on their ability to practice evidence-based medicine and use resources appropriately with consideration of economic factors and time constraints ( Christmas *et al.*, 2008). This review identified no article on the use of visual arts to teach clinical excellence in the skillful negotiation of the health care system domain of clinical excellence.

### Knowledge

Outstanding knowledge and lifelong learning are central to clinical excellence ( Christmas *et al.*, 2008). This review identified one article describing the use of visual arts to teach the knowledge domain of clinical excellence ( Finn, White and Abdelbagi, 2011). This article describes a one-hour UME curriculum that employed body painting as a creative method for teaching anatomy ( Finn, White and Abdelbagi, 2011). Medical students painted each other’s body surfaces to facilitate the learning of spatial relations of underlying anatomy and of clinical signs. The authors suggest that this method of teaching encourages active learning, appeals to all learning styles, and improves knowledge retention.

### Scholarly approach to clinical practice

This clinical excellence domain describes physicians who apply evidence thoughtfully to patient care decisions, and who are committed to improving patient care systems and disseminating clinical knowledge ( Christmas *et al.*, 2008). This review identified no study describing the use of visual arts to teach the scholarly approach to clinical practice domain of clinical excellence.

### Exhibiting a passion for patient care

Clinically excellent physicians must have a passion for, enthusiasm about, and enjoyment of clinical medicine ( Christmas *et al.*, 2008). Although this review did not identify a study that explicitly articulated the use of visual arts to teach the exhibiting a passion for patient care clinical excellence domain, all of the 15 identified studies imply that creative approaches to teaching medicine, including the incorporation of the visual arts, can help foster interest in and a passion for patient care, which may not be evoked by traditional ways of teaching.

## Discussion

Each article identified by this review describes a curriculum that uses the visual arts to teach at least one of the following domains of clinical excellence: communication and interpersonal skills, humanism and professionalism, diagnostic acumen, and knowledge. No article describes a curriculum to teach the skillful negotiation of the health care system, scholarly approach to clinical practice, and exhibiting a passion for patient care domains of clinical excellence. The lack of articles within these 3 domains may reflect an absence of such articles in the literature, or a limitation of this review’s search strategy, including search terms and limiters.

This review did identify several barriers to the incorporation of the visual arts into medical education. The introduction of new medical humanities courses can be met with resistance by learners and faculty, especially those who believe that such courses lack scientific rigor in an environment in which biomedical sciences predominate and lack supporting theorentical frameworks ( Kumagai, 2012; Mullangi, 2013; Kumagai, 2017). More rigorously designed studies that yield stronger evidence to support the value and long-term benefits of curricula that use the visual arts to enhance clinical skills are clearly needed to address this challenge ( Kumagai, 2017).

Kumagai suggests that incorporating the arts and humanities into medical education may “threaten to reproduce dominant values and perspectives” (a cultural elitism), which may in turn exclude certain individuals ( Kumagai, 2017). This review suggests that when the visual arts are incorporated into medical curricula, those learners with backgrounds in or with a particular interest in the arts tend to participate more in discussion ( Boisaubin and Winkler, 2000). Since many of the articles identified by this review describe curricula offered only as electives, the learners who chose to participate may have been more likely to have a greater interest or background in the visual arts. This may have positively skewed the results in studies that measured outcomes.

Finding skilled teachers to facilitate these curricula can also be a barrier to implementation, given that few medical schools have faculty with expertise in the medical humanities ( Boisaubin and Winkler, 2000). Conflicting views exist in the literature regarding the degree to which expertise in the visual arts is required to effectively teach arts-based medical curricula, as well as regarding how much prior knowledge is required to fully benefit learners. Some suggest that the instructor and/or learners should either have a background or expertise in the visual arts in order to effectively teach or learn from these courses ( Boisaubin and Winkler, 2000). Others suggest that a visual arts-based observation training program for medical students can be developed and implemented without specifically trained faculty ( Jasani and Saks, 2013).

A gap in the literature also exists around which institution-level barriers must be addressed in order to successfully incorporate the visual arts into medical education. For example, institutional commitment in terms of allocation of curricular time and funding may be required if visual arts-based medical curricula are to be successful ( Kumagai, 2012). Future research should also examine the degree of faculty development necessary to effectively teach these curricula, and explore the possibility of partnerships between medical schools and art institutions, and among departments of medicine, art history, and fine arts. A stronger emphasis on tailoring arts-based content to match the experience of learners is also required and should be addressed on a situation-by-situation basis.

While this review was able to identify a number of medical schools that have incorporated the visual arts into their curricula, few educators have evaluated the success of these curricula with objective learning measures. In many cases, outcomes were limited to self-report of learner reaction to the curriculum or of their learning of knowledge and skills, rather than more objective behavioral and targeted outcome measures. Most of the articles describe curricula of relatively short duration and report no long-term outcomes. Future research should focus on longitudinal studies that measure behavioral and targeted outcomes over a longer duration of learners’ medical training/practice to determine any enduring impact of the use of visual arts on the teaching of clinical excellence ( Kumagai, 2012; Jasani and Saks, 2013).

## Conclusion

This review supports the use of visual arts in medical education to teach clinical excellence in the domains of communication and interpersonal skills, professionalism and humanism, diagnostic acumen, and knowledge. Future studies specifically designed to assess the impact of the use of the visual arts to teach clinical excellence in these and other domains on physician behavior are needed.

## Take Home Messages

1. Medical educators can incorporate the viewing of representational and abstract paintings, as well as the creation of original art, into their curricula.
2. The visual arts can be used to teach: (1) communication and interpersonal skills; (professionalism and humanism; (3) diagnostic acumen; and (4) knowledge.
3. It is feasible to combine the examination of paintings with guided group discussions using visual thinking strategies and/or reflective writing, although little is known about which of these methods are most effective to teach clinical excellence.

## Notes On Contributors

**Eden Gelgoot, BSc,** is a master’s student in the Department of Psychiatry at McGill University. Her interdisciplinary educational background in the arts and sciences, as well as her interest in patient-centered health care delivery motivated her to pursue this research.

**Christine Caufield-Noll, MLIS, AHIP,** is the Manager of Library Services for the Harrison Medical Library at the Johns Hopkins Bayview Medical Center in Baltimore, Maryland.

**Margaret S. Chisolm, MD,** is an Associate Professor of and Vice Chair for Education in Psychiatry and Behavioral Sciences at the Johns Hopkins University School of Medicine in Baltimore, Maryland.

## Appendices

### Appendix 1. Highest impact medical education and general medicine journals, as ranked by InCites Journal Citation Reports

**Table:**

Journal Name	Impact Factor
**Medical Education Journals**	
Academic Medicine	4.194
Medical Education	3.369
Hematology-American Society of Hematology Education Program	3.126
Advances in Health Sciences Education	2.462
Medical Teacher	2.355
Anatomical Sciences Education	2.303
Journal of Nutrition Education and Behavior	2.253
Studies in Science Education	2.167
Journal of Surgical Education	1.950
CBE-Life Sciences Education	1.908
Chemistry Education Research and Practice	1.802
Journal of Engineering Education	1.739
Advances in Physiology Education	1.723
Nurse Education Today	1.591
Journal of School Health	1.547
Journal of Cancer Education	1.368
IEEE Transactions on Education	1.330
Physical Review Special Topics-Physics Education Research	1.316
BMC Medical Education	1.312
Journal of Chemical Education	1.225
**General Medicine Journals**	
New England Journal of Medicine	59.558
Lancet	44.002
JAMA-Journal of the American Medical Association	37.684
BMJ-British Medical Journal	19.697
Annals of Internal Medicine	16.593
JAMA Internal Medicine	14.000
PLOS Medicine	13.585
BMC Medicine	8.005
Journal of Cachexia Sarcopenia and Muscle	7.883
Journal of Internal Medicine	7.803
Canadian Medical Association Journal	6.724
Cochrane Database of Systematic Reviews	6.103
Mayo Clinic Proceedings	5.920
American Journal of Medicine	5.610
Annals of Family Medicine	5.087
Translational Research	4.557
American Journal of Preventitive Medicine	4.465
Annals of Medicine	3.763
Deutsches Arzteblatt International	3.738
Palliative Medicine	3.685

## Declarations

Dr. Chisolm is a member of the Johns Hopkins Miller Coulson Academy of Clinical Excellence.
