# Supplementary material for: Using the visual arts to teach clinical excellence
Source: MedEdPublish (2016). 2018 Jul 16;7:143. [Version 1] doi: 10.15694/mep.2018.0000143.1 (PMC10701836; doi:10.15694/mep.2018.0000143.1)
Supplement: Peer review history

# Reviewer report: Reviewer response for version 1

**DOI:** 10.21956/mep.19610.r29147

**Published:** 2018-07-18

**Authors:** Jonathan McFarland

**Affiliations:** 1 Sechenov University, Moscow

This review has been migrated. The reviewer awarded 5 stars out of 5 I read this article with growing interest, for as Trevor Gibbs mentioned in another review, I completely agree that we need to go beyond the “feel-good factor” of teaching the humanities. This fascinating article starts us on the journey of explaining how to implement the Humanities (in this case the visual arts) into the medical curriculum, which is crucial now. For me, of particular interest was Table 1, which is a summary of the articles identified on the use of visual arts in the teaching of clinical excellence, as it is very interesting to observe which paintings have been used at different institutions, what tasks the students were made to make and for what reasons. For example, students at a workshop in Israel had to investigate the Dr-patient relationship using Luke Filde’s and Picasso’s famous paintings, The Doctor and Science and Charity, both by reflecting their own experience with patients but also by going into the paintings and personifying one of the characters. A brilliant and beautiful idea. This is just one example that this paper unearths.So, for many reasons, I would wholeheartedly recommend it for all those who are interested in exploring how to bring the Humanities back into the medical curriculum, and why. Nevertheless, as the authors themselves comment in their conclusion further research designed to specifically evaluate the impact of the visual arts on clinical excellence outcomes is still needed. The journey has begun but there is still some way to go. Thank you.

Reviewer Expertise:

NA

No decision status is available

# Reviewer report: Reviewer response for version 1

**DOI:** 10.21956/mep.19610.r29144

**Published:** 2018-07-16

**Authors:** Trevor Gibbs

**Affiliations:** 1 AMEE

This review has been migrated. The reviewer awarded 5 stars out of 5 There is often a feel-good factor associated with teaching the Humanities - it feels right to explore in-depth the classical painting, to uncover feelings within a piece of prose or to contemplate the words uttered by the actors on stage or in a film. We can, as educationalists, sit back in the warmth of this feeling or we can move from the art to the science and begin to ask the more serious questions of research. To sit back and enjoy will not advance the humanities in the eyes of the sceptics, we need to begin to ask the why, how and so-what questions; why do we need the Humanities, how can we introduce them into healthcare education and most importantly, so what- do they make any difference. This very well written and well researched paper begins to answer these questions and demonstrates that there is already a body of literature that can demonstrate the "power" of teaching the humanities. I learned a lot from this paper and its references and would highly recommend it to all those who are thinking about putting a humanities course within their curriculum.

Reviewer Expertise:

NA

No decision status is available

# Reviewer report: Reviewer response for version 1

**DOI:** 10.21956/mep.19610.r29145

**Published:** 2018-07-16

**Authors:** Kathleen Leedham-Green

**Affiliations:** 1 King's College London / Imperial College London

This review has been migrated. The reviewer awarded 4 stars out of 5 This is a helpful review in an emergent field in medical education. It is well written and presented with clarity. It assesses papers that evaluate visual arts in medical education programmes against published criteria for clinical excellence and Kirkpatrick's levels of evaluation. To improve this paper, I would have appreciated an explanation of why they chose these particular criteria for clinical excellence, and more emphasis on their methods for finding/including papers. I remain unconvinced that their stated search would have uncovered all the relevant papers in this field. Several medical schools include humanities programmes that include visual arts alongside other media such as poetry, literature, film etc. Should these have been included? If not, why not. For systematic reviews on niche subjects (where there are perhaps less than 20 papers in existence) I would have appreciated more than one approach to finding papers, for example including a snowballing approach (searching up and down reference lists from the papers they identified in the original search). Finally, I particularly liked the analysis across the themes for clinical excellence, it gave a clear picture of the strengths of the visual arts in general and each programme in particular.

Reviewer Expertise:

NA

No decision status is available

# Reviewer report: Reviewer response for version 1

**DOI:** 10.21956/mep.19610.r29146

**Published:** 2018-07-16

**Authors:** P Ravi Shankar

**Affiliations:** 1 American International Medical University

This review has been migrated. The reviewer awarded 5 stars out of 5 I read with great interest this review article on the use of the visual arts to teach clinical excellence. The methodology for the selection of the studies included in this review has been comprehensively described and the authors provide the salient details of the various studies included. I and my colleagues have been using paintings for over ten years first in Nepal and later in the Caribbean. Western paintings are more readily available compared to paintings from developing countries. Students in Nepal were able to relate to paintings better when compared to literature from a western context. Some of the situations depicted in paintings are universal while others are more culture specific. The Caribbean student body is very diverse with students from North America, Africa and Asia. Many North American students are of Asian or African descent. With increasing access to technology students have access to visual arts from a number of countries. However, in many countries admission to medical school is restricted to students with a science background and these students often lack knowledge of and an appreciation of the arts. One of the challenges with regard to the medical humanities is obtaining information about their long-term impact. The subject has been well established in developed nations and studies on long term impact are beginning to emerge. Methodological and other challenges, however, remain. Obtaining resources and curricular time for the medical humanities continues to be a challenge in developing nations. The literature review was carried out in May 2017 and was limited to the PubMed and the ERIC databases. Also articles published in high impact journals were considered. As the authors mention this may have resulted in studies and initiatives not being included in this review. Despite problems with quality and there being no specific criteria for inclusion of journals, the Google Scholar database provides access to a wide range of the published literature and could be considered. I do recognize that this may require more debate. The article is well-written and will be of broad interest to medical educators.

Reviewer Expertise:

NA

No decision status is available
